# The fungicidal effectiveness of 2-Chloro-3-hydrazinylquinoxaline, a newly developed quinoxaline derivative, against Candida species

**DOI:** 10.1371/journal.pone.0303373

**Published:** 2024-05-10

**Authors:** Abdelbagi Alfadil, Karem A. Ibrahem, Mohammad W. Alrabia, Jawahir A. Mokhtar, Hafsa Ahmed

**Affiliations:** 1 Faculty of Medicine, Department of Clinical Microbiology and Immunology, King Abdulaziz University, Jeddah, Saudi Arabia; 2 Center of Research Excellence for Drug Research and Pharmaceutical Industries, King Abdulaziz University, Jeddah, Saudi Arabia; 3 King Abdulaziz Hospital, Jeddah, Saudi Arabia; University of Jeddah, SAUDI ARABIA

## Abstract

**Background:**

Candida represents a prevalent fungal infection, notable for its substantial implications on morbidity and mortality rates. In the landscape of prospective treatments, quinoxaline derivatives emerge as a category of compact compounds exhibiting notable potential in addressing infections. These derivatives showcase promising antimicrobial efficacy coupled with favorable pharmacokinetic and safety characteristics.

**Aims:**

The central aim of this investigation was to examine the antifungal characteristics of 2-Chloro-3-hydrazinylquinoxaline against diverse strains of *Candida* and *Aspergillus in vitro*. Additionally, we endeavored to assess the *in vivo* efficacy of 2-Chloro-3-hydrazinylquinoxaline using a murine model for oral candidiasis induced by *C*. *albicans* cells ATCC 10231.

**Results:**

2-Chloro-3-hydrazinylquinoxaline demonstrated noteworthy effectiveness when tested against various reference strains of *Candida* species. It exhibited heightened efficacy, particularly against *Candida krusei* isolates. However, its performance against *Candida albicans*, *Candida tropicalis*, *Candida glabrata*, *Candida parapsilosis*, and *Candida auris* isolates exhibited variability. Notably, 2-Chloro-3-hydrazinylquinoxaline manifests variable efficacy against *Aspergillus fumigatus*, *Aspergillus niger*, *Aspergillus terreus* and *Aspergillus flavus* and no effect against *Aspergillus brasiliensis*. In a murine model, 2-Chloro-3-hydrazinylquinoxaline exhibited significant efficacy in combating the *C*. *albicans* cells ATCC 10231 strain, underscoring its potential as a viable treatment option.

**Conclusion:**

2-Chloro-3-hydrazinylquinoxaline has demonstrated substantial potential in effectively addressing various *Candida* and *Aspergillus* species, showcasing dual attributes of antifungal and anti-inflammatory properties. However, to attain a more comprehensive understanding of its therapeutic capabilities, further investigations, incorporating additional tests and experiments, are imperative.

## Introduction

In critically ill patients, infections caused by *Candida* spp. stand as a prominent contributor to both morbidity and mortality [1]. These infections manifest in diverse clinical presentations, encompassing intra-abdominal candidiasis, bloodstream infections, superficial candidiasis, and deep-seated infections [2]. Over recent decades, the incidence of *Candida* spp. infections have seen a gradual rise, primarily attributed to the heightened prevalence of invasive medical procedures and the escalating utilization of broad-spectrum antimicrobials [3].

The commensal human pathogen *Candida albicans* is undergoing an evolving mechanism of drug resistance, presenting a continuous challenge. Over time, *Candida* species have developed various strategies to counteract the effects of diverse drug classes, posing a significant threat to human health [4]. The slow pace of developing new antibiotics by pharmaceutical companies is primarily attributed to challenges like resistance, regulatory hurdles, and financial constraints [5]. Consequently, addressing this issue necessitates innovative methods [6]. One such approach involves repurposing existing antibiotics through synergy, while another entails the identification of small-molecule inhibitors capable of enhancing the efficacy of other drugs [7]. These strategies aim to navigate the complexities of drug resistance in *Candida* species and offer potential solutions to combat this formidable challenge [3].

Quinoxaline derivatives constitute a distinct category of organic compounds featuring a quinoxaline ring structure within their chemical composition. The unique characteristics and potential applications of these derivatives are intricately linked to their specific chemical structures [8]. Ongoing research endeavors are dedicated to exploring novel quinoxaline derivatives and discerning their potential applications across diverse fields [9]. This sustained scientific interest is attributed to the versatile nature of these compounds, prompting continuous investigations to unveil their varied properties and potential uses. As researchers delve into the exploration of new derivatives, the landscape of quinoxaline compounds remains an evolving area of scientific curiosity, promising insights that may extend into a range of scientific and practical domains [10].

Quinoxaline derivatives have exhibited notable efficacy in combating bacterial and fungal infections, demonstrating effectiveness against pathogens such as *E*. *cloacae* and *C*. *albicans*. The intriguing aspect is that certain quinoxaline derivatives not only showcase inhibitory properties but also exhibit bactericidal and fungicidal activities [11]. This promising observation serves as a catalyst, prompting a comprehensive investigation into the efficacy of 2-Chloro-3-hydrazinylquinoxaline. The intention is to delve into a thorough assessment, both in vitro and in vivo, utilizing a mice model. This multifaceted approach seeks to unravel the potential of 2-Chloro-3-hydrazinylquinoxaline in combating microbial infections, drawing insights from the compound’s performance against specific bacterial and fungal strains. The exploration encompasses a dual perspective, combining *in vitro* studies to scrutinize molecular interactions and *in vivo* experiments utilizing a mice model to simulate a more holistic physiological context, thereby laying the foundation for a nuanced understanding of 2-Chloro-3-hydrazinylquinoxaline’s antimicrobial effectiveness.

## Materials and methods

The material employed in the study comprised 2-Chloro-3-hydrazinylquinoxaline, procured from Sigma Aldrich, and utilized without further purification. To create a stock solution of 2-Chloro-3-hydrazinylquinoxaline, 100% dimethyl sulfoxide (DMSO) from Sigma Aldrich-D8418, Taufkirchen, Germany, was employed. Subsequently, the working solution of 2-Chloro-3-hydrazinylquinoxaline was diluted in RPMI-1640 medium from Gibco, Maryland, United States of America, ensuring a final DMSO concentration of below 5%.

### Fungal species, media, and growth condition

In the *in vitro* investigations, a comprehensive set of twenty fungal reference strains was utilized, covering a diverse spectrum of pathogenic *Candida* species, including *Candida albicans*, *Candida krusei*, *Candida tropicalis*, *Candida parapsilosis*, *Candida auris*, *Candida glabrata*, as well as *Aspergillus fumigatus*, *Aspergillus niger*, *Aspergillus flavus*, *Aspergillus brasiliensis*, and *Aspergillus terreus*. For the in vivo experiments, a reference strain, *C*. *albicans* ATCC 10231, *obtained from the American* Type Culture Collection, was employed. These strains were meticulously preserved at -80°C, utilizing a 20% glycerol solution in Sabouraud’s dextrose broth for optimal viability. Cultivation of the cells involved streaking them on Sabouraud’s dextrose agar and subsequent incubation at 37°C for 36 hours. Clinical isolates utilized in this study were sourced from the Department of Medical Laboratory Technology at the Faculty of Applied Medical Sciences, King Abdulaziz University, Jeddah, Saudi Arabia. The sample collection adhered strictly to ethical guidelines and research protocols established by the Faculty of Applied Medical Sciences at King Abdulaziz University, as indicated by the Ethics Committee number 38-712-456. Given that the clinical isolates were procured as part of routine hospital laboratory procedures, the ethics committee granted an exemption from the requirement for informed consent for this research.

### Formulating a gel preparation of 2-Chloro-3-hydrazinylquinoxaline

For the preparation of a 1% hydrogel containing 2-Chloro-3-hydrazinylquinoxaline, an initial step involved introducing 2 grams of hydroxypropyl methylcellulose (HPMC) powder (Sigma Aldrich-H7509, Taufkirchen, Germany) into a suitable beaker. Subsequently, 50 ml of purified hot water was added, initiating a stirring process, and allowing the mixture to soak. Further enhancement of the gel formulation involved the addition of 10 ml of glycerol (Sigma Aldrich-G5516, Taufkirchen, Germany), with the soaking process persisting. The beaker was then covered and left undisturbed for a duration of 24 hours at room temperature, facilitating the formation of the gel.

To achieve a concentration of 1% w/w for the 2-Chloro-3-hydrazinylquinoxaline hydrogel, a separate solution was prepared by dissolving 1 gram of 2-Chloro-3-hydrazinylquinoxaline in 5 ml of 99% methanol and 35 ml of distilled water. The resultant drug solution in methanol was gradually introduced to 45 grams of plain HPMC gel, with a continuous and gentle mixing process to attain a homogenous gel consistency. The final gel formulation was thoughtfully stored in appropriate amber glass jars at 4 °C, ensuring protection against exposure to light [12].

### *In vitro* Susceptibility of 2-Chloro-3-hydrazinylquinoxaline

In this study, we assessed the antifungal efficacy of 2-Chloro-3-hydrazinylquinoxaline through in vitro examinations involving 20 reference strains. To establish the minimum inhibitory concentration (MIC), we adhered to the protocols outlined by the Clinical and Laboratory Standards Institute (CLSI). The process commenced by preparing an inoculum containing 7×10^3^ colony-forming units per ml (CFU/ml) derived from a 36-hour culture grown on Sabouraud Dextrose Agar, cultivated at 37°C. In a 96-well microtiter plate, 100 μl of RPMI-1640 medium was dispensed into each well. 2-Chloro-3-hydrazinylquinoxaline, initially at a concentration of 3 mg/mL, was added to the second column of the plate (200 μl per well). Subsequent columns underwent a two-fold serial dilution, resulting in a range of 2-Chloro-3-hydrazinylquinoxaline concentrations from 64 to 0.06 μg/mL.

Following this, the prepared inoculum was added at 100 μl per well, starting from the second column and progressing to the last column, which served as the positive control, containing only the inoculum. The first column represented the negative control and contained wells with RPMI-1640 medium only. Subsequently, the plates were incubated at 37ºC for a period of 24–36 hours. This testing procedure was conducted in duplicate on the same day and repeated in triplicate over the course of two weeks [13,14].

### The quantification of specific inflammatory markers using an enzyme-linked immunosorbent assay (ELISA)

In the evaluation of inflammation, we utilized ELISA immunoassay kits, specifically opting for the ELISA inos kit. This kit featured ELISA plates with pre-coated monoclonal antibodies meticulously designed to target the specific cytokine under examination. This approach followed a quantitative sandwich enzyme immunoassay technique known for its high precision.

To ensure precise measurements of the selected inflammatory marker, a meticulous procedure was adhered to. Standards and test samples were introduced into the individual wells of the ELISA plate. Subsequent to this, any unbound substances were thoroughly eliminated through a series of washing steps utilizing a wash buffer to remove any extraneous materials. Following the wash steps, an enzyme-linked polyclonal antibody, specifically crafted to bind to the cytokine of interest, was introduced into the wells. This step aimed to enhance assay specificity by binding exclusively to the target marker. Post the addition of the enzyme-linked antibody reagent, further meticulous washing steps were carried out to eliminate any antibodies that had not bound to the target cytokine.

The final phase involved introducing a substrate solution into the wells, initiating a color development reaction. The intensity of the resulting color was directly proportional to the quantity of the cytokine of interest that had successfully bound during the assay. To quantitatively assess this color intensity, a microplate reader was employed, ensuring precise and accurate determination of the levels of the specific inflammatory marker under investigation. This comprehensive process facilitated the rigorous quantification of the chosen marker, providing reliable and valuable data for inflammation assessment [14].

### Animal testing

For this investigation, we utilized male BALB/c mice aged between six and eight weeks, procured from the Faculty of Pharmacy at King Abdulaziz University’s animal house. These mice were granted unrestricted access to both food and water and were carefully monitored for a period of 30 days before the initiation of the experiment. The animal experiments involved a total of 32 male mice, categorized into four groups. Each group was further subdivided into two shoebox cages, accommodating 4–5 mice per cage. This subdivision strategy was implemented to minimize aggression and enhance the overall well-being of the animals, in accordance with recommendations from the National Research Council (US) Institute for Laboratory Animal Research in 2004.

The environmental conditions for the animal cages included a temperature-controlled setting, maintaining a constant temperature of 23 ± 2 °C, and a 12-hour light/dark cycle. Mice that either survived until the conclusion of the experiment or were in a moribund state underwent humane euthanasia. Euthanasia was performed by administering an overdose of a combination of ketamine and xylazine through intraperitoneal injection, followed by cervical dislocation.

The four groups were structured as follows:

1. Group 1: Uninfected control group (n = 8).
2. Group 2: Stress-exposed group, which received an inoculum but no additional treatment (n = 8).
3. Group 3: Mice that received 2-Chloro-3-hydrazinylquinoxaline at a concentration of 0.02 mg/ml along with a *C*. *albicans* ATCC 10231 inoculum (n = 8).
4. Group 4: Mice that received 2-Chloro-3-hydrazinylquinoxaline at a concentration of 0.2 mg/ml in conjunction with a *C*. *albicans* ATCC 10231 inoculum (n = 8).

All animal experiments strictly adhered to the animal care and use guidelines approved by King Abdulaziz University. The animal study protocol received approval from the Institutional Animal Care at King Abdulaziz University, aligning with the guidelines established by the Canadian Council on Animal Care (CCAC) [15].

### Animal preparation and oral infection

To instigate oral candidiasis in the mice, a specific treatment and infection protocol were implemented. The animals underwent immunosuppression through two subcutaneous injections of prednisolone (Sigma Aldrich-P6004, Taufkirchen, Germany) at a dosage of 100 mg/kg. These injections were administered one day prior to and three days following the infection with *Candida albicans* ATCC 10231. Additionally, tetracycline hydrochloride (Sigma Aldrich-T7660, Taufkirchen, Germany) was introduced into the mice’s drinking water at a concentration of 0.9 mg/ml, starting one day before the infection. Anesthesia was induced through intramuscular injections of 2 mg/ml chlorpromazine chloride (Sigma), with 50 μl administered on each femur.

To initiate oral infections, small cotton pads (baby cotton buds from Johnson & Johnson) were immersed in a cell suspension of *C*. *albicans* ATCC 10231 containing 2.0 x 10^8^ viable cells per milliliter. These saturated cotton pads were then used to gently swab the entire oral cavity of the anesthetized mice. The daily assessment of infection severity relied on monitoring the presence and intensity of whitish, curd-like patches observed on the surface of the tongue [16].

### Assessment of the progression of infections

In this experimental framework, we identified day 7 as a pivotal time point for evaluating the infection’s extent. On this critical day, groups of mice were anesthetized and humanely sacrificed to conduct a thorough assessment of tongue lesions, a key indicator of infection progression. To meticulously gauge the impact of the infection, we employed a macroscopic approach, assigning scores ranging from 0 to 4. These scores considered both the extent and severity of the whitish, curd-like patches on the tongue’s surface, serving as visible indicators of infection progression.

The scoring criteria were thoughtfully designed as follows:

- A score of 0 denoted the tongue in its normal, uninfected state.
- A score of 1 was assigned when white patches covered less than 20% of the tongue’s surface, indicating a relatively mild infection.
- For a score of 2, we considered cases where white patches encompassed more than 20% but less than 90% of the tongue’s surface, indicating a moderate level of infection.
- A score of 3 was given when the white patches extended to more than 90% of the tongue’s surface, signaling a substantial infection.
- Finally, a score of 4 was reserved for the most severe cases, where thick white patches resembling pseudomembranes enveloped more than 91% of the tongue’s surface, indicating an advanced and extensive infection.

These detailed scoring criteria provided a robust framework for the comprehensive evaluation of infection severity, enabling precise measurement of tongue lesion progression and facilitating a thorough assessment of the experimental outcomes [16].

### Antifungal treatment

At the 72-hour mark following the initial infection, we systematically grouped 32 animals into two distinct categories to examine the potential effects of our treatment approach. These groups underwent therapeutic intervention with the application of 0.5 mL of 2-Chloro-3-hydrazinylquinoxaline. This application was precisely executed 3 hours after the initial inoculation, a crucial time point in the infection timeline. The treatment plan involved the daily application of 2-Chloro-3-hydrazinylquinoxaline to the oral cavity for eight consecutive days, spanning from day 0 to day 7 post-infection. To ensure comprehensive coverage of the entire oral cavity, including the tongue, buccal mucosa, and soft palate, the topical agent was directly administered using a cotton swab. This meticulous application guaranteed thorough distribution and contact with the affected areas.

For the control group, consisting of infected animals without any treatment, we administered 0.5 mL of sterile saline. This saline solution, containing 0.8% agar, was orally administered once a day to serve as a control condition, allowing us to assess the impact of our treatment. In addition to the experimental and control groups, we established a negative control group. This group included animals that were immunosuppressed but not subjected to the fungal infection. Their immunosuppressed status aimed to simulate a condition rendering them susceptible to infection. The assessment of antifungal efficacy in this negative control group was conducted on day 3 post-infection, providing valuable insights into the treatment’s effectiveness under immunosuppressed conditions. This comprehensive experimental design enabled us to thoroughly investigate the impact of 2-Chloro-3-hydrazinylquinoxaline on fungal infection and provided a solid foundation for assessing its antifungal potential under diverse conditions [16].

### Study of histopathological changes

On the 7th day following the administration of the final dose of either the antifungal agent or saline solution, the humane euthanasia of the animals was carried out via an intraperitoneal injection of a ketamine-xylazine cocktail, followed by cervical dislocation. Subsequently, the tongues were delicately extracted and fully immersed in Bouin solution for a minimum of 48 hours to ensure optimal fixation. Following fixation, the tongues underwent further processing, involving collection, fixation in a 20% formalin solution, and eventual embedding in paraffin. The paraffin-embedded tongues were meticulously sectioned into thin 5-μm sections using a Leica microtome (Leica Microsystems Inc., Buffalo, NY, USA). These sections were then subjected to staining with hematoxylin and eosin (H&E) as well as periodic acid–Schiff (PAS) stains. The purpose of these stains was to facilitate a comprehensive examination under a light microscope (Olympus Optical Co., Ltd., Japan). This histopathological analysis aimed to discern and evaluate any histological changes and, notably, to detect the presence of fungi within the tissues. This detailed procedure was implemented to ensure a thorough histopathological examination, allowing for a comprehensive analysis of the effects of the antifungal agent and the potential presence of fungal elements in the tongue tissues of the euthanized animals [16].

### Statistical analysis

Statistical analysis in this study utilized the GraphPad Prism software, a widely acknowledged tool for such purposes. Each experiment underwent careful repetition at least twice to ensure the robustness and reliability of the results. Subsequently, mean values were meticulously calculated to offer a representative summary of the data.

To identify statistically significant differences and enable meaningful comparisons among the experimental groups, we employed a stringent unpaired t-test, a statistically recognized method known for its precision and accuracy. This approach allowed for a quantitative evaluation of data variations with a high degree of confidence. A significance level of 0.05, a conventional and widely accepted threshold, was applied to determine the statistical significance of the findings. Consistent with established conventions, p-values less than or equal to 0.05 were considered indicative of significant results. For clarity, p-values were explicitly denoted using a standardized notation: *p<0.05, **p<0.01, ***p<0.001, and ****p<0.0001, representing varying degrees of statistical significance. For a more comprehensive understanding of the statistical analyses conducted for each figure, interested readers can refer to the detailed descriptions provided within the respective figure legends. These descriptions offer a transparent and thorough account of the analytical procedures employed in this study.

## Results

### The effectiveness of 2-Chloro-3-hydrazinylquinoxaline in a laboratory setting

The antifungal efficacy of 2-Chloro-3-hydrazinylquinoxaline was evaluated *in vitro* through the broth microdilution method. Minimum Inhibitory Concentration (MIC) values for 2-Chloro-3-hydrazinylquinoxaline were determined and are presented in Table 1. Following a 24-hour incubation period, the compound demonstrated increased effectiveness, especially against *Candida krusei* isolates. However, its efficacy varied when tested against *Candida albicans*, *Candida tropicalis*, *Candida glabrata*, *Candida parapsilosis*, and *Candida auris* isolates. These observed variations in efficacy against distinct *Candida* species underscore the compound’s potential selectivity and specificity in its antifungal activity. Notably, 2-Chloro-3-hydrazinylquinoxaline exhibited commendable variable efficacy against *Aspergillus fumigatus*, *Aspergillus niger*, *Aspergillus flavus* and *Aspergillus terreus*, and displaying limited activity against *Aspergillus brasiliensis*. This specificity in antifungal properties is evident in the compound’s performance.

**Table 1:** The *in vitro* activity of 2-Chloro-3-hydrazinylquinoxaline against different *Candida* and *Aspergillus* specious. Conc, concentration.

No. of Sample	Isolate	2-Chloro-3-hydrazinylquinoxaline	
		Conc μg/ml 24h	Conc μg/ml 48h
1	*Candida albicans* ATCC 90028	>64	>64
2	*Candida albicans* ATCC MYA 573	32	>64
3	*Candida albicans* MMX 7424	64	64
4	*Candida tropicalis* ATCC 90874	>64	>64
5	*Candida tropicalis* MMX 7525	64	64
6	*Candida glabrata* ATCC 90030	64	64
7	*Candida glabrata* MMX 7285	64	64
8	*Candida parapsilosis* ATCC 22019	>64	>64
9	*Candida krusei* ATCC 6258	16	64
10	*Candida auris* MMX 9867	64	64
11	*Aspergillus fumigatus* ATCC MYA 3626	64	64
12	*Aspergillus fumigatus* ATCC 204305	64	64
13	*Aspergillus fumigatus* ATCC MYA 4609	64	64
14	*Aspergillus fumigatus* ATCC 32820	16	16
15	*Aspergillus niger* ATCC 9508	64	64
16	*Aspergillus niger* MMX 5953	64	64
17	*Aspergillus flavus* ATCC 22546	>64	>64
18	*Aspergillus flavus* ATCC 64025	64	64
19	*Aspergillus brasiliensis* ATCC 16404	>64	>64
20	*Aspergillus terreus* ATCC 3628	64	64

These findings offer a nuanced insight into the antifungal profile of 2-Chloro-3-hydrazinylquinoxaline, highlighting its potential effectiveness against specific *Candida* strains and certain *Aspergillus* spp while indicating limitations in its activity against other *Aspergillus* species.

#### Macroscopic assessment of the oral mucosa in mice

**Fig 1:**
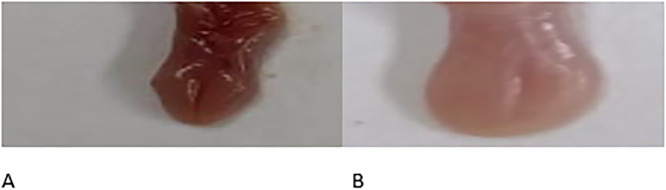
A visual examination was conducted to assess standard lesions characterized by white patches on the tongues of mice affected by oral candidiasis. The examination categorized tongue conditions into two distinct groups: (A) representing the normal tongue appearance following treatment with 2-Chloro-3-hydrazinylquinoxaline and (B) illustrating tongues featuring characteristic white patches. This visual assessment aimed to provide a detailed depiction of the observed oral lesions and distinguish the visual differences between the unaffected and affected groups, contributing to a thorough understanding of the oral status following the induction of oral candidiasis.

To evaluate the oral status post-*Candida* infection induction, a thorough examination of oral symptoms in infected mice was conducted. This assessment included assigning scores based on both the size and intensity of white patches observed on the mice’s tongues. Fig 1 visually illustrates this examination, highlighting distinctive oral lesions in mice with oral candidiasis compared to those in the treated group. The visual inspection in Fig 1 vividly displays characteristic lesions, featuring prominent white patches on the tongues of mice affected by oral candidiasis, contrasting with the tongue appearance of the treated group. The notable difference in tongue conditions between the infected and treated groups is evident, emphasizing the discernible effects of the treatment regimen. This method not only provides a qualitative visual representation of observed changes but also employs a scoring system to quantitatively capture the extent of these alterations, offering a comprehensive understanding of the treatment’s impact on the oral status of the mice.

### Changes in the histopathological features of the oral mucosa in mice

In our investigation into the clinical manifestations accompanying infections in the oral mucosa induced by *C*. *albicans*, a detailed examination was conducted using histopathological slides derived from tissue samples. Oral candidiasis in mice, a fungal infection predominantly linked to *Candida* species, exhibits distinctive histological features evident in sagittal sections of the mice’s tongues. These characteristic tissue lesions present a nuanced pathological spectrum, prominently featuring epidermal hyperplasia (acanthosis). Additionally, our observations extended to the identification of both moderate and severe inflammatory cells and pseudohyphae, providing insights into the inflammatory response and the severity of the infection.

**Fig 2:**
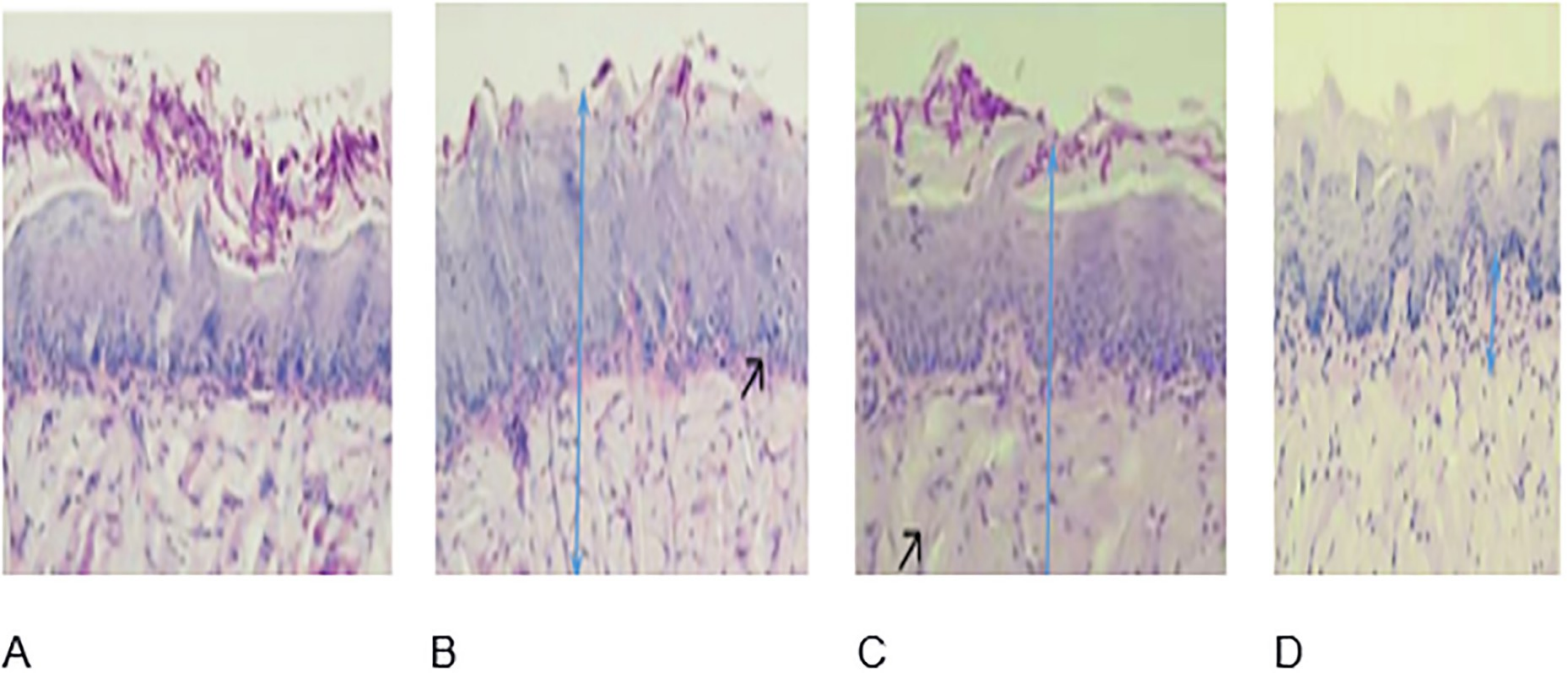
Tongue biopsies were classified into the following groups for detailed histological analysis. A. Control Negative (Uninfected Group): Serving as the baseline reference for normal histological features in the absence of infection. B. Candida Infected Group: Comprising mice with induced Candida infection in the oral cavity, with a focus on characteristic changes associated with Candida-induced lesions. C. Treated with Low Dose (0.02 mg/ml). D. Treated with High Dose (0.2 mg/ml): Mice in this group received treatment with a higher dose (0.2 mg/ml) of the experimental agent, aiming to evaluate the dose-dependent effects on tissue integrity and infection-related lesions.

Notably, our investigation delved into the impact of administering 2-Chloro-3-hydrazinylquinoxaline to mice at various concentrations. The histopathological slides revealed significant improvements in tissue conditions following this treatment. These enhancements were evident in the form of a reduction in epithelial hyperplasia, the restoration of a normal basal layer, and the recovery from hyperplasia-induced alterations in inflammatory cells and pseudohyphae. This observed improvement underscores the potential therapeutic efficacy of 2-Chloro-3-hydrazinylquinoxaline in ameliorating histopathological changes associated with oral candidiasis, providing insights into its effects on tissue integrity and the inflammatory milieu (Fig 2).

### The *in vivo* effectiveness of 2-Chloro-3-hydrazinylquinoxaline against *Candida* was evaluated using a mice oral infection model

In a murine model, we evaluated the *in vivo* efficacy of 2-Chloro-3-hydrazinylquinoxaline against *C*. *albicans* ATCC 10231, consistent with prior *in vitro* findings. Administering 2-Chloro-3-hydrazinylquinoxaline at different concentrations (0.02 and 0.2 mg/ml) significantly reduced TNF-a levels compared to the untreated infected mice group, with a p-value below 0.05. This underscores the potential anti-inflammatory effects of 2-Chloro-3-hydrazinylquinoxaline *in vivo*, indicating its ability to modulate immune responses during *C*. *albicans* infection. Moreover, introducing 2-Chloro-3-hydrazinylquinoxaline at varying concentrations (0.02 and 0.2 mg/ml) led to a marked decrease in IL6 levels compared to the untreated infected mice group, with a statistically significant p-value below 0.05. Interestingly, this reduction in IL6 was more pronounced with higher doses of 2-Chloro-3-hydrazinylquinoxaline. Similar trends were observed with IL-B1, where substantial decreases were noted with both concentrations (0.02 and 0.2 mg/ml) compared to the untreated infected mice group, yielding a p-value below 0.05. This suggests a consistent and dose-dependent impact of 2-Chloro-3-hydrazinylquinoxaline on reducing IL6 and IL-B1 levels, indicating its potential as an anti-inflammatory agent.

**Fig 3:**
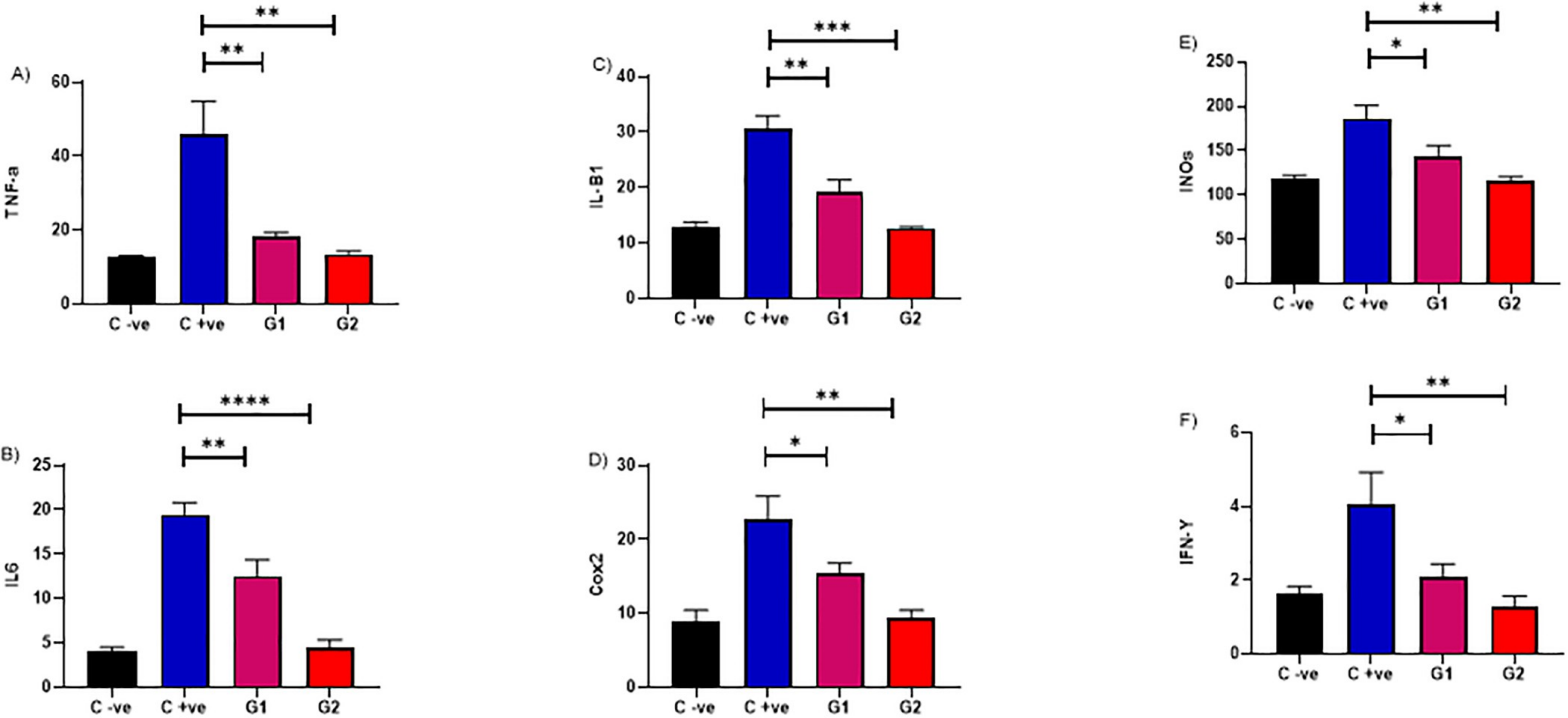
Depicts the in vivo efficacy of 2-Chloro-3-hydrazinylquinoxaline in a mice model against C. albicans ATCC 10231. Administration of 2-Chloro-3-hydrazinylquinoxaline at concentrations of 0.02 and 0.2 mg/ml resulted in a significant reduction in inflammatory markers compared to the untreated infected mice group, with a p-value below 0.05. G1 represents the group receiving 0.02 mg/ml, and G2 represents the group receiving 0.2 mg/ml. The assessed inflammatory markers include TNF-a, IL6, IL-B1, Cox2, INOs, and IFN-γ.

Furthermore, applying 2-Chloro-3-hydrazinylquinoxaline at diverse concentrations (0.02 and 0.2 mg/ml) resulted in a significant decline in Cox2 levels compared to the untreated infected mice group, with a p-value below 0.05. Notably, the reduction in Cox2 was more pronounced at higher doses of 2-Chloro-3-hydrazinylquinoxaline. Upon administration of 2-Chloro-3-hydrazinylquinoxaline at varying concentrations (0.02 and 0.2 mg/ml), a significant reduction in INOs levels was noted compared to the untreated infected mice group, with a p-value below 0.05. Additionally, when 2-Chloro-3-hydrazinylquinoxaline was administered at varying concentrations (0.02 and 0.2 mg/ml), a noteworthy decrease in IFN gama levels was observed compared to the untreated infected mice group, with the p-value falling below 0.05. Interestingly, the reduction in IFN gama levels remained consistent between the two concentration levels. This observation suggests that 2-Chloro-3-hydrazinylquinoxaline may exhibit a dual effect, concurrently manifesting anti-inflammatory and antifungal properties (Fig 3).

## Discussion

*Candida* infections pose an escalating challenge, particularly with the emergence of drug-resistant strains [17]. The process of drug discovery is fraught with various challenges, encompassing ethical, economic, and time-related factors [18]. A comprehensive understanding of these challenges is crucial for the development of new and efficient drugs. A promising strategy to address the issue of antimicrobial resistance (AMR) involves exploring new indications for established drugs and presenting a novel and optimistic approach [7]. This alternative circumvents the need to create entirely new drugs, a process known for its costliness and time-intensive nature [7]. To our knowledge, our study marks the initial demonstration that quinoxaline derivatives can effectively eliminate *Candida* infection in mice.

The administered quinoxaline derivatives demonstrated efficacy *in vitro* as well as in treating mice, as evidenced by the restoration of inflammatory markers to levels closely resembling those in the control group. Furthermore, our findings demonstrate a noticeable distinction in tongue conditions between the infected and treated groups, with compelling evidence showcasing a near restoration of the tongue to its normal condition in mice. Recent studies reveal that a novel set of 4-alkoxy-6,9-dichloro triazolo[4,3-a]quinoxalines exhibit promising inhibitory properties against pro-inflammatory cytokines TNF-α and IL-6. This observation suggests a potential link between the effectiveness of quinoxalines as anti-inflammatory agents and their ability to target these specific cytokines [19]. This suggests that the administered medicine is effective in eradicating *Candida* infection in the mouse model, showcasing a promising avenue for combating drug-resistant strains.

2-Chloro-3-hydrazinylquinoxaline presents a potential remedy for combating *Candida* infections, particularly in the context of drug resistance. The challenge of drug resistance poses a hindrance to the timely development of novel drugs [20]. Discovering a drug with a new indication, specifically antifungal activity, could alleviate the shortfall in drug discovery [6]. However, it is imperative to ensure that this drug adheres to rigorous standards of efficacy, safety, and accessibility while addressing the complexities associated with *Candida* infections [6]. Furthermore, this drug has demonstrated several advantages, including anti-inflammatory and antifungal activities, prompting us to delve deeper into its characterization.

*In vitro* studies have demonstrated the notable efficacy of quinoxaline derivatives against fungal infections caused by *C*. *albicans*. Our findings indicate that the effectiveness observed *in vitro* may extend to *in vivo* conditions, aligning with prior research. Additionally, a study by Vanessa Kaplum et al highlighted the favorable in vivo efficacy of a 2,3-diarylsubstituted quinoxaline derivative in a murine cutaneous leishmaniasis model. Notably, this derivative exhibited low cytotoxicity [21]. While our results are consistent with these findings, it is imperative to assess the toxicity profile of the 2-Chloro-3-hydrazinylquinoxaline comprehensively. Additionally, it is imperative to conduct a resistance assay to evaluate the potential for bacteria to develop resistance to the combination of penicillin and 2-Chloro-3-hydrazinylquinoxaline. Gaining insight into the resistance potential is fundamental for ensuring the sustained effectiveness of this treatment strategy in the long term.

Quinoxaline possesses the ability to hinder DNA synthesis and promote the generation of reactive oxygen species (ROS). The heightened ROS production disrupts specific cellular processes, leading to cell death. These distinct antimicrobial mechanisms may contribute to elucidating its effectiveness against fungal infections [22].

It is crucial to acknowledge that, despite the promise shown in our studies, the development of antifungal agents is a complex process that demands thorough testing in preclinical models and, ultimately, clinical trials to ensure safety and efficacy in humans [23]. Therefore, additional tests are necessary to validate our findings. These tests include pharmacodynamic and pharmacokinetic assessments, which evaluate the drug’s impact on the body, its mechanism of action, potential interactions with other drugs, as well as its absorption, distribution, metabolism, and excretion [24]. This information constitutes essential components of drug development and evaluation, playing a pivotal role in the comprehensive assessment of the safety and efficacy of a new drug.

## Conclusion

For the first time, our study marks the inaugural demonstration of the remarkable efficacy of 2-Chloro-3-hydrazinylquinoxaline in both *in vivo* and *in vitro* settings. The compound, showing substantial promise, emerges as a potential solution for combating various Candida species and A. fumigatus. Nevertheless, it is imperative to underscore that the journey toward the advancement of this agent necessitates further research and development endeavours to fully harness its therapeutic potential.

## Abbreviations

*A. brasiliensis*: *Aspergillus brasiliensis*
*A. flavus*: *Aspergillus flavus*
*A. fumigatus*: *Aspergillus fumigatus*
*A. niger*: *Aspergillus niger*
*A. terreus*: *Aspergillus terreus*
AMR: Antimicrobial resistance
*C. albicans*: *Candida albicans*
*C. glabrata*: *Candida glabrata*
*C. krusei*: *Candida krusei*
*C. parapsilosis*: *Candida parapsilosis*
*C. tropicalis*: *Candida tropicalis*
CCAC: Canadian Council on Animal Care
CLSI: Clinical and Laboratory Standards Institute
COX: Cyclooxygenase
DMQ: Dimethylquinoxaline
DMSO: Dimethyl sulfoxide
IL-1β: Interleukin-1β
IL-6: Interleukin-6
iNOS: Nitric oxide synthase
MIC: Minimum inhibitory concentrations
ROS: Reactive oxygen species
RPMI: Roswell Park Memorial Institute
TNF-α: Tumor necrosis factor-alpha
