# Correction: The fungicidal effectiveness of 2-Chloro-3-hydrazinylquinoxaline, a newly developed quinoxaline derivative, against Candida species

**DOI:** 10.1371/journal.pone.0309235

**Published:** 2024-08-15

**Authors:** Abdelbagi Alfadil, Karem A. Ibrahem, Mohammad W. Alrabia, Jawahir A. Mokhtar, Hafsa Ahmed

In the Funding statement, the grant number from the Instructional Improvement Fund is listed incorrectly. The correct grant number is: IFPIP:1456-140-1443-260678.
